# Functional Diversity of the Microbial Community in Healthy Subjects and Periodontitis Patients Based on Sole Carbon Source Utilization

**DOI:** 10.1371/journal.pone.0091977

**Published:** 2014-03-14

**Authors:** Yifei Zhang, Yunfei Zheng, Jianwei Hu, Ning Du, Feng Chen

**Affiliations:** 1 Central Laboratory, School of Stomatology, Peking University, Beijing, P. R. China; 2 Department of Periodontology, School of Stomatology, Peking University, Beijing, P. R. China; University of Toronto, Canada

## Abstract

Chronic periodontitis is one of the most common forms of biofilm-induced diseases. Most of the recent studies were focus on the dental plaque microbial diversity and microbiomes. However, analyzing bacterial diversity at the taxonomic level alone limits deeper comprehension of the ecological relevance of the community. In this study, we compared the metabolic functional diversity of the microbial community in healthy subjects and periodontitis patients in a creative way—to assess the sole carbon source utilization using Biolog assay, which was first applied on oral micro-ecology assessment. Pattern analyses of 95-sole carbon sources catabolism provide a community-level phenotypic profile of the microbial community from different habitats. We found that the microbial community in the periodontitis group had greater metabolic activity compared to the microbial community in the healthy group. Differences in the metabolism of specific carbohydrates (e.g. β-methyl-D-glucoside, stachyose, maltose, D-mannose, β-methyl-D-glucoside and pyruvic acid) were observed between the healthy and periodontitis groups. Subjects from the healthy and periodontitis groups could be well distinguished by cluster and principle component analyses according to the utilization of discriminate carbon sources. Our results indicate significant difference in microbial functional diversity between healthy subjects and periodontitis patients. We also found Biolog technology is effective to further our understanding of community structure as a composite of functional abilities, and it enables the identification of ecologically relevant functional differences among oral microbial communities.

## Introduction

Periodontal disease is one of the most common adult diseases, leading to disorders of the supporting structures of the teeth, including the gingivae, periodontal ligaments, and supporting alveolar bone. The accumulation of plaque around the gingival margin triggers gingivitis [1], [2]. Once the homeostasis of microbial diversity within the plaque is lost, gingivitis leads to periodontitis [3], [4].

Attempts have been made to compare the microbial diversity in patients with periodontitis and healthy subjects [4]–[7]. Socransky *et al.* (1998) reported that the “orange complex,” consisting of Gram-negative, anaerobic species such as *Prevotella intermedia* and *Fusobacterium nucleatum*, was associated with periodontitis, whereas the “red complex,” consisting of periodontal pathogens such as *Porphyromonas gingivalis*, *Tannerella forsythia*, and *Treponema denticola*, is detected as the disease worsens [3], [8]; Using the Human Oral Microbe Identification Microarray, Colombo *et al.* (2009) detected more species in patients with periodontal disease compared to those without disease.[9]; Using 16S pyrosequencing, Griffen *et al.* (2012) found that community diversity was higher in periodontal disease subjects [10]; Abusleme *et al.* (2013) reported that the shifts in community structure from health to periodontitis are characterized by the emergence of newly dominant taxa without replacement of primary health-associated species [11]. However, these studies merely focused on the taxonomic structure of communities, and thus provided limited insight into the ecological relevance of microbial community structure. Because changes in bacterial types do not necessarily change the function of a community [12], the phenetic status of the microbial community should be considered when the microflora are studied.

Analyses of phenetic characteristics such as microbial metabolism allow for deeper insight into microbial community structure. First, metabolic processes represent a key component in determining the virulence properties of oral pathogens [13], [14]. Furthermore, the development of food chains between bacteria and endogenous nutrient metabolism would enhance the diversity of the microflora, which plays a key role in maintaining homeostasis within a microbial community [15]. For example, frequent carbohydrate consumption increases the levels of mutans streptococci and lactobacilli but decreases *Streptococcus sanguinis* levels [16], Aggressive periodontitis appears to be associated with a loss of colonization by *S. sanguinis* [17]. Grenier and Mayrand (1986) also reported that the nutritional relationships among oral bacteria could explain the mechanisms favoring bacterial succession in periodontal sites [18].

The sole carbon source utilization (SCSU) patterns of microbial samples determined using the Biolog assay (Biolog Inc., Hayward, CA, USA) [19] could be used as a functionally based measure for classifying heterotrophic microbial communities. The Biolog assay for community analysis involves outgrowth of the entire microbiota ecosystem on multiple carbon substrates. Each well contains tetrazolium violet and a minimal amount of proprietary growth media. Color produced from the reduction of tetrazolium violet is used as an indicator of respiration. Commercially available microplates allow for the simultaneous testing of 95 separate carbon sources such that the metabolic response patterns of microbial communities from different habitats can be compared [20]–[23]. Anderson *et al.* first analyzed the metabolic similarity of experimental dental plaque biofilms [24]. However, the metabolism or activities of the microflora in periodontitis patients and healthy subjects has not been reported.

Here we used Biolog technology to compare the microbial functional diversity between patients with chronic periodontitis and healthy controls to further our knowledge of the ecological basis of periodontal disease.

## Materials and Methods

### Subjects and sample collection

Plaque samples were collected from 11 patients who had been diagnosed with generalized chronic periodontitis (according to the international Classification of Periodontal Diseases in 1999: more than 30% of sites with a pocket depth >4 mm, inter-proximal attachment loss of >3 mm, bleeding on probing, and radiographic evidence of alveolar bone loss; course >6 weeks) and 12 controls that had been defined as “periodontally healthy” by a licensed periodontist. The following inclusion criteria were used: at least 18 years of age, a minimum of six natural teeth in every quadrant, an absence of other oral diseases such as caries or mucosal disease, and good systemic health. The exclusion criteria were professional periodontal therapy in the 6 months before enrollment, antibiotic use for any purpose within 1 month before entering the study, and smoking. For inclusion in the healthy group, subjects were required to have no clinical signs of inflammation, including redness, swelling, or bleeding on probing, and no pockets with a probing depth >3 mm.

Supragingival and subgingival plaque around the gingival margin was collected 2–3 h after a meal. To avoid salivary contamination, the sample collection sites (≤1 mm above the gingival margin for supragingival plaque collection, and ≤3 mm below the gingival margin for subgingival plaque collection) were isolated with cotton rolls and gently air-dried. Pooled plaque samples were carefully taken from 16 teeth (2 premolars and 2 molars in each quadrant, but not wisdom teeth) of each subject using a scaler and stored in phosphate-buffered saline (PBS; 0.01 M, pH = 7.2–7.4) (Biotop, Huangshang City, China) on ice.

The study protocol was approved by the Institutional Review Board (IRB) of Peking University School and Hospital of Stomatology (Beijing, China) (approval number: PKUSSIRB-2012063). Participants have provided their written informed consent to participate in this study, and the consent procedure was also approved by the IRB of Peking University School and Hospital of Stomatology.

#### Biolog assays

The collected plaque samples were re-suspended in 11 ml of PBS (0.01 M, pH = 7.2–7.4) and vortexed thoroughly for 60 s. Each plaque suspension was inoculated into Biolog anaerobic-negative (AN) microplates (Biolog Inc.) at 100 μl per well. The Biolog AN plates contained 95 sole carbon sources and a blank well with water only (Table S1 in File S1). The initial optical densities (ODs) of the plaque suspensions were measured before inoculation.

The plates were incubated in a 5% CO_2_ incubator at 37°C for up to 4 days. The OD at 590 nm (OD_590_) in each well was recorded every 24 h using a Biolog microstation and associated software (Biolog OmniLog version 4.1).

### Data analyses

Reactions were interpreted as positive or negative using Biolog OmniLog software. Positive wells found in at least 50% of subjects in the healthy and periodontitis groups were defined as core utilized carbon sources.

The overall metabolic activity for a microbial community in the Biolog plates was expressed as average well color development (AWCD) and calculated as follows:
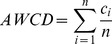



Where C is the corrected OD value (obtained by subtracting the OD of the control well from that of each experimental well to correct for the background activity) in each well and n is the number of substrates (n = 95). If the result was negative, the OD would be deemed to be zero. An independent *t*-test was performed on the measurement data to compare the mean differences in AWCD between the periodontal disease group and healthy group over 4 days.

Richness and evenness were determined using the Shannon index [25], while dominance was determined using the Simpson index [26]. To avoid negative values with the Simpson index, the control-corrected OD was multiplied by 1000.

To avoid artificial differences (differences caused by varying initial OD values of the plaque suspensions, rather than by the pattern of carbon source consumption) between the communities, we standardized the corrected OD value as proposed previously [19], [27] by dividing each corrected OD value by the AWCD of the plate. Differently exploited carbon sources were measured based on standardized OD values using independent *t*-tests and Pearson's correlation [27] (SPSS Statistics version 17.0).

The relationships between the healthy and periodontitis groups based on discriminative carbon sources were determined using cluster analyses (Cluster 3.0, Java TreeView, version 1.1.3) and principle component analyses (PCAs) (Canoco, version 4.5).

## Results

### Functional diversity in the healthy and periodontitis groups

**Figure 1:**
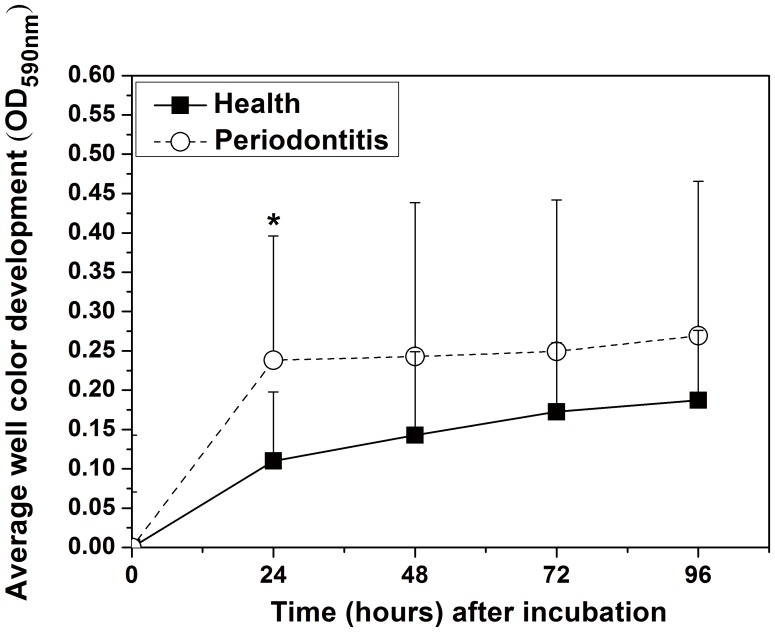
Color development (AWCD) with incubation time in the healthy and periodontitis groups (* indicates a significant difference).

The demographic and clinical characteristics of the subjects are described in Table 1. The initial OD value of the inoculum was higher in the periodontitis group compared to the healthy group, which reflects the greater amount of accumulated plaque in the periodontitis group. Compared to the healthy group, the periodontitis group yielded greater metabolic responses. Significant differences in the overall rate of color development (AWCD) between the healthy and periodontitis groups were noted during the linear increase stage of inoculation (i.e., the first 24 h in Fig. 1). The curves then presented an asymptotic nature during later incubation periods. No significant difference was found between the Shannon indices of diversity between the healthy and periodontitis groups (*p*>0.05, *t*-test; Table 2). The periodontitis group had a significantly higher Simpson diversity index value than the healthy group at 72 and 96 h after incubation (*p*<0.05, *t-*test) (Table 2).

**Table 1:** Demographic and clinical characteristics of the subjects.

Parameter	Healthy subjects	Periodontitis patients	*p-*value
No. of subjects	12	11	
Age (years)	34.6±7.1	34.9±12.5	NS^a^
Missing teeth	None	None	—
Smoker	None	None	—
Women (%)	50	54.5	NS^b^
Percentage of sites with a PD (%):			
>6 mm	0	27.3	
4–6 mm	0	54.5	
<4 mm	100	18.2	
OD_630_ of plaque suspension	0.04±0.028	0.08±0.03	0.003^a^

a Independent *t*-tests

b Chi-square tests

NS: not significant

PD: pocket depth

Quantitative data are presented as the mean±standard deviation (SD); categorical data are presented as percentages.

**Table 2:** Diversity indices of the periodontally healthy and diseased groups during incubation (mean±SD, *p*<0.05).

Index		Healthy subjects	Periodontitis patients	*p-*value
Shannon	24 h	3.98±0.45	4.08±0.23	0.52
	48 h	3.84±0.37	3.95±0.36	0.44
	72 h	3.77±0.35	3.99±0.40	0.16
	96 h	3.81±0.31	3.95±0.32	0.31
Simpson	24 h	47±22	51±12	0.27
	48 h	37±18	47±18	0.10
	72 h	32±13	50±19	**0.025**
	96 h	33±12	45±15	**0.025**

### Core positive carbon sources utilized by the microbial community in healthy subjects and periodontitis patients

**Figure 2:**
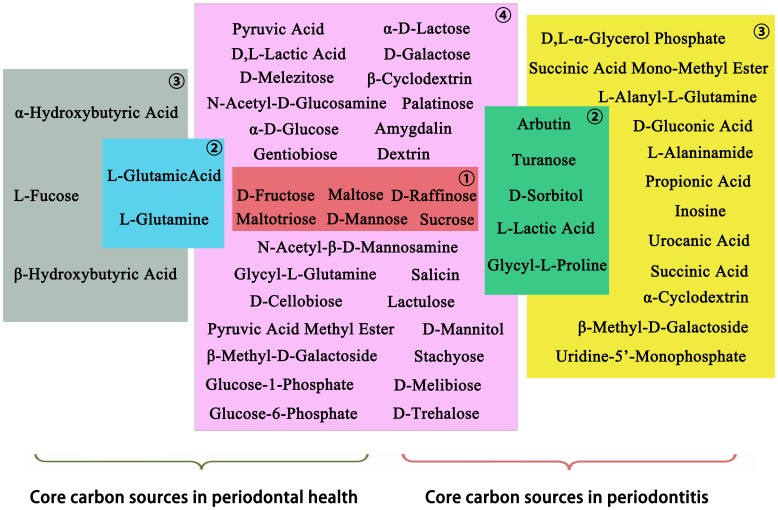
Core positive carbon sources in the healthy subjects and periodontitis patients. Inner box (numbered with 1), positive carbon sources found in all subjects (100%); middle boxes (numbered with 2), present in 71–99% of subjects from each group (H: healthy, P: periodontitis); outer boxes (numbered with 3), present in 50–70% of subjects from the healthy and periodontitis groups; middle box (numbered with 4), positive carbon sources present in at least 50% of subjects in both the healthy and periodontitis groups.

Despite varying intensity levels, a total of 31 carbon sources were positive in most subjects (≥50%) in the healthy or periodontitis group after 96 h incubation, which was defined as core positive carbon sources (Fig. 2). Six of these were found in all subjects: D-fructose, maltose, D-raffinose, maltotriose, D-mannose, and sucrose. Another five sources were present in most healthy subjects, whereas the remaining 17 were preferentially associated with periodontitis (Fig. 2). The periodontitis group harbored more core positive carbon sources than the healthy group (Fig. 2).

### Correlation between healthy status and carbon source utilization patterns

**Figure 3:**
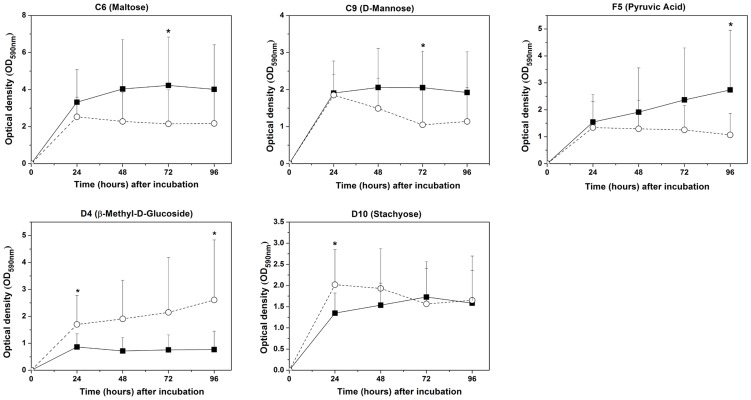
Catabolic kinetics according to inoculation time for discriminative core positive carbon sources (* indicates a significant difference) in the healthy (—▪—) and periodontitis (--○--) groups.

Analyses of 95-sole carbon source utilization patterns showed 14, 8, 11, and 8 significantly different (*p*<0.05) carbon sources between the two groups at 24, 48, 72, and 96 h, respectively (Table S2 in File S1). Of these, five core positive carbon sources were identified at different time points (Fig. 3): β-methyl-D-glucoside and stachyose were identified at 24 h, maltose and D-mannose were identified at 72 h, and β-methyl-D-glucoside and pyruvic acid at 96 h.

**Figure 4:**
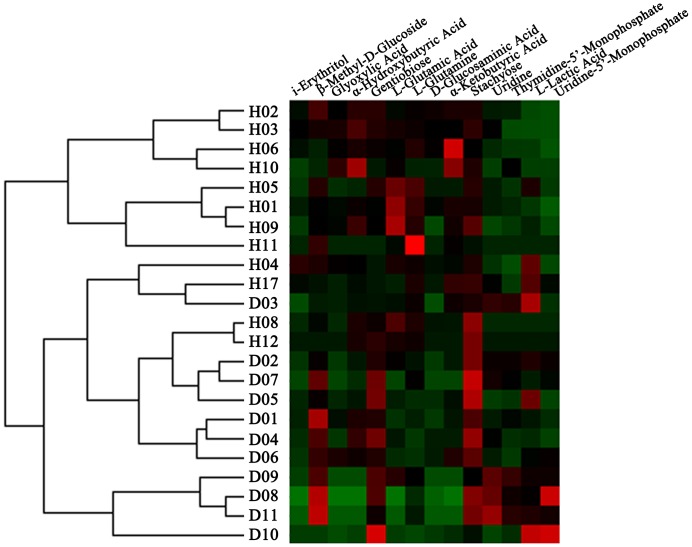
Cluster analyses of 23 clinical plaque samples from healthy (H) and diseased (D) subjects based on standardized OD values of the discriminative carbon sources at 24 h.

To identify correlations between the pattern of carbon source consumption and healthy status, cluster analyses of 23 subjects from each group were performed based on 14 discriminative carbon sources, which distinguished the healthy group (H) from the diseased group (D) at 24 h (Fig. 4). Our results suggest that the 23 samples could be classified into two groups: 8 of the 12 samples from the healthy group were classified as cluster one, while the 11 samples from the diseased group were classified as cluster two, which coincided with the clinical classification according to the diagnosis. However, certain pairs of samples from different groups were closely associated, such as samples H07 and D04 (Fig. 4). Additionally, the data indicate that some of the samples in the healthy group were similar to those in the diseased group (e.g., H04, H07, H08, and H12) (Fig. 4).

**Figure 5:**
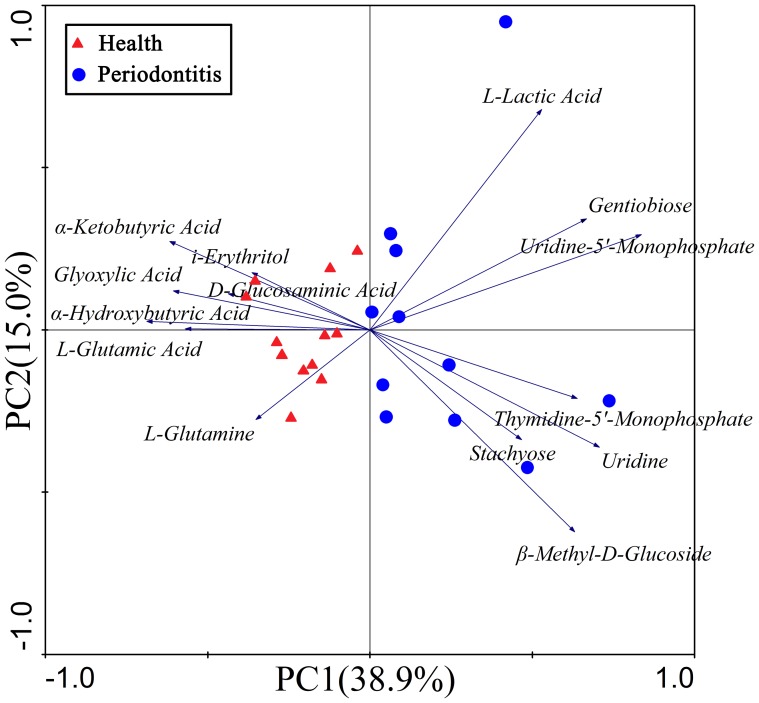
Ordination biplot of principal component analyses of the substrate utilization patterns of the microbial communities in the healthy and periodontitis groups using Biolog AN plate assays. Arrows indicate the directions and relative importance (arrow lengths) of the 14 substrates variables.

PCAs were performed to identify those carbon sources with key roles in difference between groups at 24 h. We maintained four PCs in the subsequent analysis because they accounted for more than 75% of the total eigenvalue sum (Table 3). Our PCA plots show that PC1 and PC2 accounted for 38.9 and 15% of the total variance, respectively (Fig. 5). Subsequent *t-*tests for the PCs indicated significant differences (*p*<0.05) between the two groups on PC1 (Table 3) and identified the carbon sources that best described these differences: E5 (glyoxylic acid), E6 (α-hydroxybutyric acid), E9 (α-ketobutyric acid), and B9 (D-glucosaminic acid) were identified most positively by their PC1 scores, whereas H12 (uridine-5′-monophosphate), H10 (uridine), and H11 (thymidine-5′-monophosphate) were identified most negatively by their PC1 scores (Table 4). The utilization patterns of each microbial community were compared by principal component analysis (PCAs) of the 24 h absorption data. Microbial community samples from different subjects served as objects and the absorbance values of carbon source utilization as variables (Fig.5). It is possible to identify the substrates which contribute to the separation of each sample plot on the ordination biplot (Fig. 5).

**Table 3:** *t*-tests for PCs extracted from 14 discriminative carbon sources at 24 h (*p*<0.05).

	Healthy subjects	Periodontitis patients	*p-*value
PC1	1.69±1.13	−1.85±1.74	<0.001
PC2	−2.98±1.89	0.33±0.78	0.32
PC3	0.23±0.92	−0.26±1.58	0.38
PC4	0.10±1.03	−0.11±1.43	0.68

**Table 4:** Substrates with high correlation coefficients (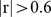) for PC1 in a PCA of substrate utilization patterns among microbial communities from periodontally healthy and diseased groups.

	r	*p-*value
E5 (Glyoxylic acid)	0.78	<0.001
E6 (α-Hydroxybutyric acid)	0.76	<0.001
E9 (α-Ketobutyric acid)	0.73	<0.001
H12 (Uridine-5′-monophosphate)	−0.72	<0.001
B9 (D-Glucosaminic acid)	0.66	0.001
H10 (Uridine)	−0.65	0.001
H11 (Thymidine-5′-monophosphate)	−0.64	0.001

## Discussion

Plaque around the gingival margin, with its toxic products, stimulates gingival tissue directly and leads to tissue destruction, likely causing periodontal disease [28]. Thus, in this study, we focused on plaque around the gingival margin from patients with periodontitis and orally healthy individuals to determine whether there were functional differences between the two groups. Because many of the anaerobic bacteria isolated from deep subgingival sites are proteolytic and asaccharolytic [28], deeper sampling (>3 mm) would not be optimal because these organisms may not be responsive to the substrates.

Comparisons of overall color development between groups were dependent on both the density and composition of the inoculum: samples with a denser microbial community would produce more effective reactions on the premise that a larger percentage of microorganisms are able to utilize the substrate. However, in this study, we did not achieve equivalent inoculum densities by dilution or concentration of the samples despite performing functional tests on the original community. The microbial community in the periodontitis patients was more metabolically active than that in the orally healthy individuals, indicating that both the amount and composition of plaque around the gingival margin accounted for the functional diversity. Thus, we confirmed the importance of plaque control in the initial therapy of periodontal disease. In addition, we normalized the raw color response data to the AWCD to account for different inoculum densities.

Analyses of sole carbon source consumption levels indicated differences in the metabolism of specific carbohydrates between the healthy and periodontitis groups. Differences in carbon source utilization are related to the color response in a given well, which is related to the number of microorganisms that are able to utilize the substrate within that well. Increased color development in certain response wells suggests that the inoculum contained a larger number of microorganisms able to utilize the substrate. The lag phase in color development in a single well (Fig. 2) indicates that a longer period of growth would produce a sufficient density of stained cells; a smaller percentage of microorganisms in the inoculum able to utilize the substrate may be reflected at later incubations times. This could explain the variety of discriminating sole carbon sources at different incubation times (Table S2 in File S1). Moreover, the patterns are a reflection of functional potential rather than *in situ* functional ability because growth plays an important role in this assay.

Cluster analyses were carried out based on the utilization of the discriminative carbon sources at 24 h because the most significant differences in sole carbon source consumption levels were obtained at this phase (linear increase; Fig. 2). In the present study, cluster analyses were used to describe the associations between healthy status and sole carbon source consumption level. Here, we hypothesized that closely associated samples possess similar community-level metabolic functions. This classification further improved the metabolic discrimination of specific carbohydrates between healthy subjects and periodontitis patients, and periodontal health or disease could be distinguished using Biolog AN plates. Furthermore, certain samples in the healthy group were closely associated with those in the disease group. This suggests that the disease-associated metabolic pattern was also found in the healthy subjects. The presence of disease-associated function might be prognostic for future disease in the absence of disease symptoms.

PCA revealed the carbon sources that contributed most to the diversity; it is well established that the biomass and diversity of the microbial community in supragingival and subgingival plaque around the gingival margin are distinguished by the consumption of substrates. In our study, the periodontitis patients resulted in a larger biomass accumulation of the microbial community (indicated by initial OD value) and remarkable changes in its diversity (indicated by AWCD). The close links between the biomass and diversity of the microbial community and the periodontal health status can be reflected by the carbon sources metabolism in plaque samples.

Because oral microbial communities may be dominated by <1000 species-level taxa [10], [29], evaluation of the dominant functional characteristics may be more accurate and efficient for classifying and characterizing microbial community structure [9]. Our findings suggest that periodontitis communities result from ecological shifts in community structure [11] as well as shifts in metabolic function. Despite differences in plaque amounts between the healthy subjects and periodontitis patients, most carbon sources utilized in the periodontitis group were also utilized by the healthy group, albeit at varying sole carbon source consumption levels. However, more carbon sources dominated in the periodontitis group compared to the healthy group. The core microbiome has already been defined in healthy individuals and periodontitis patients [6], [11]; nevertheless, it may not be adequate to interpret functional shifts. Different bacterial types would have the same function; for example, both *Streptococcus* spp. and *Neisseria* spp. can utilize sucrose. Conversely, different species in the same genera could have different metabolic functions. *Streptococcus mutans* is able to utilize mannitol but *Streptococcus mitis* cannot. Clearly, the concept of core microbial ecology may be better defined with community function measurements rather than taxonomic structure or membership.

The Shannon index is used extensively in the measurement of taxonomic diversity and community structure [30]. However, using the Shannon index to characterize functional microbial diversity may be questionable [31]. In this study, richness was defined as the total number of carbon substrates utilized while evenness was defined as the equability of substrate utilization between all utilized substrates [31]. The Biolog carbon substrate richness and evenness did not vary between the healthy subjects and periodontitis patients. Other variables that can affect microbial diversity such as differences in the dominant species may have contributed to the higher Simpson index in the periodontitis group at later incubation stages, which coincided with the higher number of core positive carbon sources in the periodontitis group. Although carbon source utilization profiles are not a direct representation of bacterial growth, the Biolog substrate catabolic responses for the microbial communities exhibited a lag phase (before 24 h, data not shown), an exponential phase, and a stationary phase, similar to bacterial growth curves. This nonlinearity has important implications for the interpretation of the disparate catabolic kinetics patterns of different carbon sources (as depicted in Fig. 2) and the single fixed-time readings of community Biolog responses.

Microbial community in our oral cavity is not constant, and would be easily affected by environmental factors such as temperature, pH value and so on. Thus analyzing the microbial diversity in a fixed-time point would be a misleading in our comprehension. In our study, the changes in community metabolism according to time had been well demonstrated by Biolog assay (indicated by AWCD and Simpson index), reflecting the changes in community structure.

One limitation of Biolog technology is that it cannot detect microorganisms that do not make use of the carbon sources on the Biolog microplate. Additionally, the response to substrate catabolism requires an effective quantity and activity of the microbial community in the tested samples. Nevertheless, Biolog technology was found to be an effective assay to rapidly visualize community structure as a composite of functional abilities or potential, and it enables the identification of ecologically relevant functional differences among oral microbial communities. Our study showed a distinguishing characteristic of carbonate metabolism, which may provide a potential adjuvant method for the diagnosis of periodontitis. More importantly, certain carbohydrates should be avoided in the diet according to your suggestion, which might prevent susceptible individuals from developing periodontitis.

## Supporting Information

File S1 **Table S1 & S2.** Table S1. Carbon-sources pattern of the Biolog AN microplate. Table S2 Discriminative carbon sources between health (H) group and periodontitis group (P) based on standardized OD values (*p*<0.05).
(DOC)

## References

[1] Loe H, Theilade E, Jensen SB (1965) Experimental gingivitis in man. J Periodontol 36: 177–187. 14296927 10.1902/jop.1965.36.3.177

[2] Theilade E, Wright WH, Jensen SB, Loe H (1966) Experimental gingivitis in man. II. A longitudinal clinical and bacteriological investigation. J. Periodont. Res. 1: 1–13. 4224181 10.1111/j.1600-0765.1966.tb01842.x

[3] Socransky SS, Haffajee AD, Cugini MA, Smith C, Kent RL Jr (1998) Microbial complexes in subgingival plaque. J Clin Periodontol 25: 134–144. 9495612 10.1111/j.1600-051x.1998.tb02419.x

[4] Moore WE, Moore LV (1994) The bacteria of periodontal diseases. Periodontol 2000 5: 66–77. 9673163 10.1111/j.1600-0757.1994.tb00019.x

[5] Aas JA, Paster BJ, Stokes LN, Olsen I, Dewhirst FE (2005) Defining the normal bacterial flora of the oral cavity. J Clin Microbiol 43: 5721–5732. 16272510 10.1128/JCM.43.11.5721-5732.2005 PMC1287824

[6] Bik EM, Long CD, Armitage GC, Loomer P, Emerson J, et al (2010) Bacterial diversity in the oral cavity of 10 healthy individuals. Isme Journal 4: 962–974. 20336157 10.1038/ismej.2010.30 PMC2941673

[7] Zhang S-M, Tian F, Huang Q-F, Zhao Y-F, Guo X-K, et al (2011) Bacterial diversity of subgingival plaque in 6 healthy Chinese individuals. Exp Ther Med 2: 1023–1029. 22977615 10.3892/etm.2011.311 PMC3440835

[8] Socransky SS, Haffajee AD (2005) Periodontal microbial ecology. Periodontol 2000 38: 135–187. 15853940 10.1111/j.1600-0757.2005.00107.x

[9] Colombo APV, Boches SK, Cotton SL, Goodson JM, Kent R, et al (2009) Comparisons of Subgingival Microbial Profiles of Refractory Periodontitis, Severe Periodontitis, and Periodontal Health Using the Human Oral Microbe Identification Microarray. J Periodontol 80: 1421–1432. 19722792 10.1902/jop.2009.090185 PMC3627366

[10] Griffen AL, Beall CJ, Campbell JH, Firestone ND, Kumar PS, et al (2012) Distinct and complex bacterial profiles in human periodontitis and health revealed by 16S pyrosequencing. Isme Journal 6: 1176–1185. 22170420 10.1038/ismej.2011.191 PMC3358035

[11] Abusleme L, Dupuy AK, Dutzan N, Silva N, Burleson JA, et al (2013) The subgingival microbiome in health and periodontitis and its relationship with community biomass and inflammation. Isme Journal 7: 1016–1025. 23303375 10.1038/ismej.2012.174 PMC3635234

[12] White DC, Findlay RH (1988) Biochemical markers for measurement of predation effects on the biomass, community structure, nutritional status, and metabolic activity of microbial biofilms. Hydrobiologia 159: 119–132.

[13] Shah HN, Seddon SV, Gharbia SE (1989) Studies on the virulence properties and metabolism of pleiotropic mutants of Porphyromonas gingivalis (Bacteroides gingivalis) W50. Oral Microbiol Immunol 4: 19–23. 2628863 10.1111/j.1399-302x.1989.tb00401.x

[14] Mazumdar V, Snitkin ES, Amar S, Segre D (2009) Metabolic network model of a human oral pathogen. J Bacteriol 191: 74–90. 18931137 10.1128/JB.01123-08 PMC2612419

[15] Marsh PD (1994) Microbial ecology of dental plaque and its significance in health and disease. Adv Dent Res 8: 263–271. 7865085 10.1177/08959374940080022001

[16] Minah GE, Solomon ES, Chu K (1985) The association between dietary sucrose consumption and microbial population shifts at six oral sites in man. Arch Oral Biol 30: 397–401. 3861144 10.1016/0003-9969(85)90066-4

[17] Stingu CS, Eschrich K, Rodloff AC, Schaumann R, Jentsch H (2008) Periodontitis is associated with a loss of colonization by Streptococcus sanguinis. J Med Microbiol 57: 495–499. 18349371 10.1099/jmm.0.47649-0

[18] Grenier D, Mayrand D (1986) Nutritional relationships between oral bacteria. Infect Immun 53: 616–620. 2875029 10.1128/iai.53.3.616-620.1986 PMC260836

[19] Garland JL, Mills AL (1991) Classification and characterization of heterotrophic microbial communities on the basis of patterns of community-level sole-carbon-source utilization. Appl Environ Microbiol 57: 2351–2359. 16348543 10.1128/aem.57.8.2351-2359.1991 PMC183575

[20] Haack SK, Garchow H, Klug MJ, Forney LJ (1995) Analysis of factors affecting the accuracy, reproducibility, and interpretation of microbial community carbon source utilization patterns. Appl Environ Microbiol 61: 1458–1468. 16534996 10.1128/aem.61.4.1458-1468.1995 PMC1388414

[21] Schutter M, Dick R (2001) Shifts in substrate utilization potential and structure of soil microbial communities in response to carbon substrates. Soil Biol Biochem 33: 1481–1491.

[22] Rusznyak A, Vladar P, Molnar P, Reskone MN, Kiss G, et al (2008) Cultivable bacterial composition and BIOLOG catabolic diversity of biofilm communities developed on Phragmites australis. Aquatic Botany 88: 211–218.

[23] Fisk MC, Ruether KF, Yavitt JB (2003) Microbial activity and functional composition among northern peatland ecosystems. Soil Biol Biochem 35: 591–602.

[24] Anderson SA, Sissons CH, Coleman MJ, Wong L (2002) Application of carbon source utilization patterns to measure the metabolic similarity of complex dental plaque biofilm microcosms. Appl. Environ. Microbiol 68: 5779–5783. 12406781 10.1128/AEM.68.11.5779-5783.2002 PMC129915

[25] Staddon WJ, Duchesne LC, Trevors JT (1997) Microbial diversity and community structure of postdisturbance forest soils as determined by sole-carbon-source utilization patterns. Microbial Ecology 34: 125–130. 9230100 10.1007/s002489900042

[26] Kuhn I, Austin B, Austin DA, Blanch AR, Grimont PAD, et al (1996) Diversity of Vibrio anguillarum isolates from different geographical and biological habitats, determined by the use of a combination of eight different typing methods. Syst Appl Microbiol 19: 442–450.

[27] Glimm E, Heuer H, Engelen B, Smalla K, Backhaus H (1997) Statistical comparisons of community catabolic profiles. J Microbiol Methods 30: 71–80.

[28] Marsh PD, Martin MV (2009) Oral Microbiology. United Kingdom: Elsevier. 63 p.

[29] Dewhirst FE, Chen T, Izard J, Paster BJ, Tanner ACR, et al (2010) The Human Oral Microbiome. J Bacteriol 192: 5002–5017. 20656903 10.1128/JB.00542-10 PMC2944498

[30] Krebs CJ (1994) Ecology: The Experimental Analysis of Distribution and Abundance. New York: Harper Collins College Publishers. 514p.

[31] Derry AM, Staddon WJ, Trevors JT (1998) Functional diversity and community structure of microorganisms in uncontaminated and creosote-contaminated soils as determined by sole-carbon-source-utilization. World J Microbiol Biotechnol 14: 571–578.

