# Supplementary material for: Functional Diversity of the Microbial Community in Healthy Subjects and Periodontitis Patients Based on Sole Carbon Source Utilization
Source: PLoS One. 2014 Mar 14;9(3):e91977. doi: 10.1371/journal.pone.0091977 (PMC3954802; doi:10.1371/journal.pone.0091977)
Supplement: File S1 — Table S1 & S2. Table S1. Carbon-sources pattern of the Biolog AN microplate. Table S2 Discriminative carbon sources between health (H) group and periodontitis group (P) based on standardized OD values (p<0.05). (DOC) [file pone.0091977.s001.doc]

Supplementary Table S1 Carbon-sources pattern of the Biolog AN microplate

| A1 Water | A2  N-Acetyl-D-Galactosamine | A3  N-Acetyl-D-Glucosamine | A4  N-Acetyl-β-D-Mannosamine | A5  Adonitol | A6  Amygdalin | A7  D-Arabitol | A8  Arbutin | A9  D-Cellobiose | A10  α-Cyclodextrin | A11  ß-Cyclodextrin | A12  Dextrin |
| --- | --- | --- | --- | --- | --- | --- | --- | --- | --- | --- | --- |
| B1  Dulcitol | B2  i-Erythritol | B3  D-Fructose | B4  L-Fucose | B5  D-Galactose | B6  D-Galacturonic Acid | B7  Gentiobiose | B8  D-Gluconic Acid | B9  D-Glucosaminic Acid | B10  α-D-Glucose | B11  Glucose-1-Phosphate | B12  Glucose-6-Phosphate |
| C1  Glycerol | C2  D,L-α-Glycerol Phosphate | C3  m-Inositol | C4  α-D-Lactose | C5  Lactulose | C6  Maltose | C7  Maltotriose | C8  D-Mannitol | C9  D-Mannose | C10  D-Melezitose | C11  D-Melibiose | C12  3-Melthyl-D-Glucose |
| D1  α-Methyl-D-Galactoside | D2  ß-Methyl-D-Galactoside | D3  α-Methyl-D- Glucoside | D4  ß-Methyl-D-Glucoside | D5  Palatinose | D6  D-Raffinose | D7  L-Rhamnose | D8  Salicin | D9  D-Sorbitol | D10  Stachyose | D11  Sucrose | D12  D-Trehalose |
| E1  Turanose | E2  Acetic Acid | E3  Formic Acid | E4  Fumaric Acid | E5  Glyoxylic Acid | E6  α-Hydroxybutyric Acid | E7  ß-Hydroxybutyric Acid | E8  Itaconic | E9  α-Ketobutyric Acid | E10  α-Ketovaleric Acid | E11  D,L-Lactic Acid | E12  L-Lactic Acid |
| F1  D-Lactic Acid Methyl Ester | F2  D-Malic Acid | F3  L-Malic Acid | F4  Propionic Acid | F5  Pyruvic Acid | F6  Pyruvic Acid Methyl Ester | F7  D-Saccharic Acid | F8  Succinamic Acid | F9  α-Succinic Acid | F10  Succinic Acid Mono-Methyl Ester | F11  m-Tartaric Acid | F12  Urocanic Acid |
| G1  L-Alaninamide | G2  L-Alanine | G3  L-Alanyl-L-Glutamine | G4  L-Alanyl-L-histidine | G5  L-Alanyl-L-Threonine | G6  L-Asparagine | G7  L-Glutamic Acid | G8  L-Glutamine | G9  Glycyl-L-Aspartic Acid | G10  Glycyl-L-Glutamine | G11  Glycyl-L-methionine | G12  Glycyl-L-Proline |
| H1  L-Methionine | H2  L-Phenylalanine | H3  L-Serine | H4  L-Threonine | H5  L-Valine | H6  L-Valine plus L-Aspartic Acid | H7  2’-Deoxy Adenosine | H8  Inosine | H9  Thymidine | H10  Uridine | H11  Thymidine-5’-Monophosphate | H12  Uridine-5’-Monophosphate |

Table S2 Discriminative carbon sources between health (H) group and periodontitis group (P) based on standardized OD values (*p*＜0.05).

| Carbon sources | 24h | | 48h | | 72h | | 96h | |
| --- | --- | --- | --- | --- | --- | --- | --- | --- |
|  | H | P | H | P | H | P | H | P |
| A7 (D-Arabitol) |  |  |  |  |  | ↑ |  | ↑ |
| B1(Dulcitol) |  |  |  | ↑ |  | ↑ |  |  |
| B2 (i-Erythritol) | ↑ |  |  |  |  |  |  |  |
| B7 (Gentiobiose) |  | ↑ |  | ↑ |  | ↑ |  | ↑ |
| B9 (D-Glucosaminic Acid) | ↑ |  |  |  |  |  |  |  |
| C6 (Maltose) |  |  |  |  | ↑ |  |  |  |
| C8 (D-Mannitol) |  |  |  | ↑ |  |  |  |  |
| C9 (D-Mannose) |  |  |  |  | ↑ |  |  |  |
| D1(α-Methyl-D-Galactoside) |  |  |  | ↑ |  | ↑ |  | ↑ |
| D4 (ß-Methyl-D-Glucoside) |  | ↑ |  | ↑ |  |  |  | ↑ |
| D10 (Stachyose) |  | ↑ |  |  |  |  |  |  |
| E5 (Glyoxylic Acid) | ↑ |  |  |  |  |  |  |  |
| E6 (α-Hydroxybutyric Acid) | ↑ |  |  |  |  |  |  |  |
| E9 (α-Ketobutyric Acid) | ↑ |  | ↑ |  | ↑ |  | ↑ |  |
| E12 (L-Lactic Acid) |  | ↑ |  |  |  |  |  |  |
| F5 (Pyruvic Acid) |  |  |  |  |  |  | ↑ |  |
| F12 (Urocanic Acid) |  |  |  | ↑ |  |  |  |  |
| G1(L-Alaninamide) |  |  |  |  |  | ↑ |  | ↑ |
| G3(L-Alanyl-L-Glutamine) |  |  |  |  |  | ↑ |  | ↑ |
| G7 (L-Glutamic Acid) | ↑ |  | ↑ |  | ↑ |  |  |  |
| G8 (L-Glutamine) | ↑ |  |  |  |  |  |  |  |
| H10 (Uridine) |  | ↑ |  |  |  |  |  |  |
| H11 (Thymidine-5’-Monophosphate) |  | ↑ |  |  |  | ↑ |  |  |
| H12 (Uridine-5’-Monophosphate) |  | ↑ |  |  |  |  |  |  |

↑: the utilization is higher than the other group
